# mTOR signalling mediates the spinal osteoblast pathotype at the curve apex in adolescent idiopathic scoliosis

**DOI:** 10.1038/s41598-026-56019-1

**Published:** 2026-07-01

**Authors:** Ellie H. Northall, Thomas A. Nicholson, Jon Hazeldine, Liam M. Grover, Helen M. McGettrick, Matthew Newton Ede, Amy J. Naylor, Simon W. Jones

**Affiliations:** 1 Department of Inflammation and Ageing, College of Medicine and Health, University of Birmingham, Birmingham, B15 2TT UK; 2 NIHR Biomedical Research Centre, Birmingham, B15 2TT UK; 3 Healthcare Technologies Institute, School of Chemical Engineering, College of Engineering and Physical Sciences, University of Birmingham, Birmingham, B15 2TT UK; 4 The Royal Orthopaedic Hospital, Birmingham, B31 2AP UK

**Keywords:** Scoliosis, Bone, Osteoblasts, mTOR, Spine, Rapamycin, Cell biology, Diseases, Molecular biology

## Abstract

Adolescent idiopathic scoliosis (AIS) is associated with dysregulated bone remodelling, yet the molecular underpinnings remain unclear. To investigate site-specific osteoblast phenotypes at the spinal curve apex, we performed bulk RNA sequencing on primary matched osteoblasts isolated from the convex, concave, and non-curve regions of AIS patients. Principal component analysis revealed distinct transcriptional clustering by spinal site, independent of patient-specific factors. Differential expression analysis identified region-specific molecular profiles in convex and concave osteoblasts compared to non-curve controls, with the mTOR pathway being highlighted as one of the most dysregulated. Rapamycin, an mTOR inhibitor, reduced Alkaline phosphatase (ALP) activity, Osteoprotegerin (OPG) secretion, and mineralization, while modulating osteogenic gene expression, including sustained upregulation of *RUNX2* and *COL1A1*. In AIS patient-derived osteoblasts, rapamycin elicited pronounced inhibition of mTOR signalling and osteogenic activity in convex cells compared to control. Convex osteoblasts also showed elevated mTOR expression but reduced downstream translation-related signalling, suggesting dysregulated or uncoupled mTOR activity. Notably, mTOR expression level correlated with curve severity, reinforcing the link between mTOR dysregulation and AIS pathology. These findings identify mTOR signalling as a key regulatory pathway in AIS osteoblast dysfunction and highlight rapamycin as a potential, though complex, therapeutic candidate.

**Supplementary Information:**

The online version contains supplementary material available at https://doi.org/10.1038/s41598-026-56019-1.

## Introduction

Adolescent idiopathic scoliosis (AIS) is one of the most common forms of paediatric spinal deformities^1^. Defined as abnormal lateral curvature of the spine, it occurs during the adolescent growth spurt, usually between the ages of 10 and 18 and requires a lateral vertebral rotation (Cobb angle) of greater than 10° to meet the clinical definition^2^. It is estimated to affect 2–4% of this population^3^ and accounts for approximately 90% of idiopathic scoliosis in adolescents. AIS is a complex, multifaceted condition with a poorly understood pathophysiology; however, growing evidence suggests that intrinsic factors, particularly genetic influences, play a significant underlying role.

Individuals who have a first degree relative affected by AIS have an increased risk of developing the condition^4^, and people who inherit single nucleotide polymorphisms (SNPs) in the POC5^5–8^ and/or TTLL11^9^ genes also have an increased risk. As scoliosis is widely reported to be more prevalent^10–13^ and more severe in females than in males^14–18^, the role of hormones such as oestrogen has been investigated. To this end, it has been reported that female AIS patients exhibit lower blood oestradiol level than age-matched control females^19^, suggesting a potential hormonal contribution to the condition’s genetic susceptibility.

Increasing evidence suggests that abnormalities in spinal bone development and remodelling are a central driver of AIS pathology. Notably, AIS patients exhibit altered bone structural parameters, including lower bone mineral density (BMD)^20^ and asymmetric vertebral growth compared to age-matched healthy controls - features that may contribute directly to curve initiation and progression. Despite these observations, investigations into the underlying cellular and molecular mechanisms for these findings are limited, largely due to the ethical and logistical access challenges of obtaining spinal tissue samples from paediatric patients. Consequently, much of the current understanding is derived from animal models such as zebrafish or chickens, which although useful, do not fully replicate the human condition in terms of biomechanical environment or developmental timing. Human studies have instead focused on surrogate tissues, such as blood, or cells derived from muscle^21^ or cartilage^22^, which offer indirect insights into spinal pathology. However, a growing number of studies are now beginning to directly assess spinal osteoblasts, the drivers of bone formation, in AIS patients^23,24^, offering a more targeted approach to understanding the skeletal abnormalities that underlie this complex disorder.

Critically, we recently reported that spinal osteoblasts at the curve apex in AIS patients exhibit intrinsic functional impairment, with greater metabolic and proliferative capacity observed in osteoblasts isolated from the convex site of the curve apex, compared to concave or non-curve osteoblasts from the same patients^25^. Therefore, we hypothesised that dysregulated signalling in spinal osteoblasts at the curve apex plays a central underlying role in the development of AIS. The aim of this study was therefore to determine the cellular mediators of these dysfunctional curve apex AIS osteoblasts, such that candidate pathways and targets can be identified for subsequent therapeutic modulation.

## Results

## Spinal AIS osteoblasts at the curve apex exhibit a differential transcriptomic phenotype

**Fig. 1:**
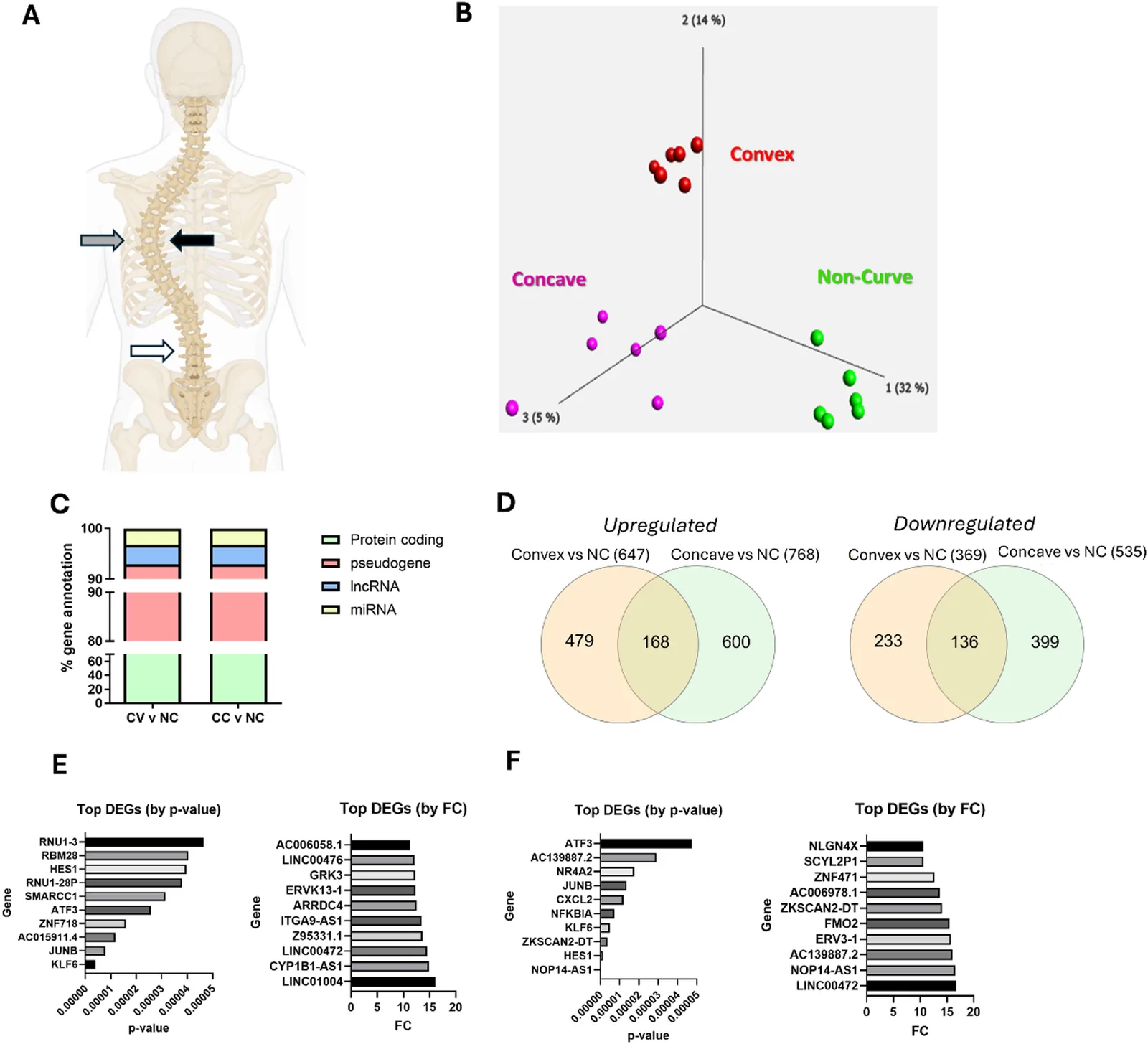
RNAseq analysis reveals distinct transcriptomic profiles between non-curve and curve apex site AIS patient spinal osteoblasts. (**A**) Schematic showing tissue collection sites at the curve apex (convex – grey arrow, concave – black arrow) and non-curve control (white arrow) (made in BioRender). (**B**) Principal component analysis of RNAseq data of *n* = 6 patients showing clustering by spinal site. (**C**) Percentages of gene transcript type identified by RNA sequencing in convex (CV) vs. non-curve (NC) and in concave (CC) vs. non-curve (NC). (**D**) Venn diagram showing the number of upregulated and downregulated differentially expressed genes (DEGs; *p* ≤ 0.05, FC ≥ ± 1.5) between convex and non-curve, and between concave and non-curve, and those shared between the 2 comparisons. (**E**) Top DEGs by p-value and fold-change (FC) for the concave vs. non-curve comparison and (**F**) for the convex vs. non-curve comparison.

Initially, we investigated whether AIS patient osteoblasts at the curve apex exhibit an intrinsic differential transcriptomic phenotype, compared to osteoblasts from a non-curve site. Osteoblasts were isolated from either side of the curve apex (convex and concave) and osteoblast from non-curve spinal facet tissue (Fig. 1A) of *n* = 6 AIS patients (see Table 1 for clinical details) and subjected to bulk RNA sequencing. Principal component analysis (PCA) of the RNA sequencing data revealed distinct clustering by spinal site (i.e. concave, convex or non-curve), independent of patient (Fig. 1B). This strikingly demonstrates that spinal site was a greater factor in the overall osteoblast gene expression phenotype than patient-specific characteristics including sex or age. Notably, convex and concave osteoblasts formed separate clusters, indicating that each side of the curve apex is associated with a distinct transcriptional identity rather than representing a uniform “curved” phenotype.

**Table 1:** Demographic information for AIS patient tissue donors used in this study.

ID	Age (years)	Sex	Height (cm)	Weight (kg)	BMI	Waist circ. (cm)	Hip circ. (cm)	Waist : Hip (ratio)	Cobb angle (°)
1*	18	M	170	54.6	18.9	74	76	0.97	54.5
2	16	F	160	71.7	28.0	97	106	0.92	50.6
3*	18	F	160	71.7	28.0	78	87	0.90	52.2
4*	18	F	162	66	25.1	86	106	0.81	43.2
5*	16	F	157	43.6	17.7	62.5	74.5	0.84	74.5
6	17	F	152	42.8	18.5	76	89	0.85	52.8
7*	17	F	171	60.2	20.6	78	95	0.82	76.2
8*	16	F	155	59.9	24.9	96	98	0.98	64.6
9	16	M	174	64.8	21.4	94	96	0.98	65.5
10	16	F	159	45.6	18.0	72	86	0.84	42.1
11	16	F	163	60	22.6	-	-	-	43.6
12	17	F	162	54	20.6	84	87	0.97	48.2
13	16	F	156	45.5	18.7	63	83	0.76	61.1
14	-	-	-	-	-	-	-	-	-
15	16	F	158	45.7	18.3	73	79	0.92	-
16	18	F	158	52.7	21.1	66	84	0.79	47.2
17	17	M	177	57.6	18.4	69	80	0.86	58.5
18	16	M	169	59.2	20.7	74	93	0.80	41.9
19	17	F	166	65.3	23.7	75	95	0.79	57.2
20	16	M	168	48.4	17.1	68	83	0.82	44.8
21	18	M	194	91.4	24.3	87	100	0.87	59.3
22	16	F	160	55.6	21.7	69	90	0.77	46.4

All details are anonymised data that were available to researchers from patients at the time of surgery. Matching tissue from each patient was collected from three sites for use in experimental analyses. A dash (-) denotes no clinical data available for the indicated variable. A star (*) denotes the subgroup of samples from which RNA sequencing was performed.

Two datasets of differentially expressed genes (DEGs) (*p* < 0.05; >1.5 fold-change) were generated from this analysis: (1) Comparison of patient-matched convex curve osteoblasts with non-curve osteoblasts; (2) Comparison of patient-matched concave curve osteoblasts with non-curve osteoblasts. Interestingly, both DEG datasets showed similar proportions of gene types including mRNAs as well as non-coding RNAs such as microRNAs, lncRNA and pseudogenes (Fig. 1C), suggesting that broad transcriptional regulation, rather than selective gene class effects, underlies the observed differences. In total, 647 upregulated DEGs were identified in the convex vs. non-curve comparison, whilst 768 upregulated DEGs identified between concave vs. non-curve, of which 168 genes (representing 26%) were common to both comparisons (Fig. 1D). Correspondingly, 369 DEGs were downregulated in the convex vs. non-curve dataset, whilst 535 DEGs were downregulated between concave vs. non-curve, of which 136 DEGS (representing 37%) were common to both comparisons. This partial overlap indicates the presence of both shared curve apex–associated transcriptional changes and region-specific molecular signatures unique to the convex or concave side.

In the concave vs. non-curve comparison, the most significant DEG by p-value was the transcription factor kruppel-like factor 6 (KLF6) (p-value 0.00000407, q-value 0.23707534) followed by transcription factor JUNB (p-value 0.00000791, q-value 0.23707534), while the DEG with the largest fold change was long intergenic non-protein coding RNA 1004 (LINC01004) (FC + 16.0985641) (Fig. 1E). In the convex vs. non-curve comparison, the most significant DEG by p-value was NOP14 Antisense RNA 1 (NOP14-AS1) (p-value 0.000000136, q-value 0.00825825) followed by the transcription factor hairy and enhancer of split-1 (HES1) (p-value 0.00000156, q-value 0.04734841), while the DEG with the largest fold change was long intergenic non-protein coding RNA 472 (LINC00472) (+ 16.77462044 FC) (Fig. 1F).

Together, these data demonstrate that osteoblasts at the AIS curve apex possess an intrinsic, spatially defined transcriptomic phenotype characterised by both shared and region-specific gene expression changes. To determine the biological significance of these transcriptional differences, we then interrogated dysregulated signalling pathways and upstream regulatory mechanisms associated with each curve region.

### Dysregulated canonical signalling pathways in curve apex osteoblasts reveals candidate upstream regulators

**Fig. 2:**
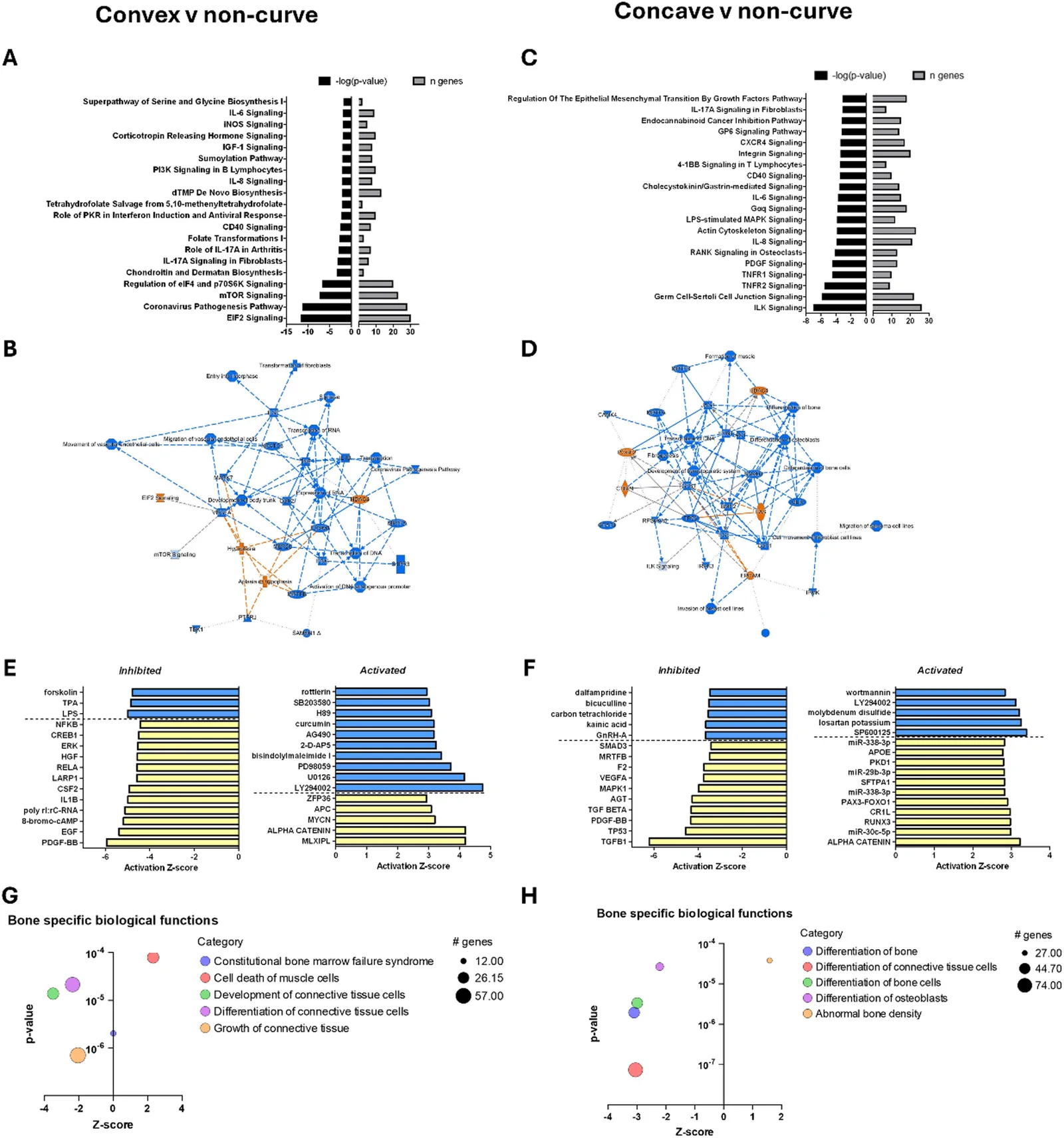
Pathway analysis highlights dysregulated signalling pathways, biological processes and upstream regulators between AIS spinal osteoblasts at the curve apex compared to non-curve. (**A**) Top canonical pathways of the convex vs. non-curve DEGs as determined using IPA, sorted by p-value with the number of genes involved in the pathway shown, with (**B**) graphical summary where blue=predicted inhibition and orange=predicted activation. (**C**) Top canonical pathways of the concave vs. non-curve DEGs, sorted by p-value with the number of genes involved in the pathway shown, with (**D**) graphical summary where blue=predicted inhibition and orange=predicted activation. (**E**) Top upstream regulators (USRs) of the convex vs. non-curve DEGs that are predicted to be either activated or inhibited. Yellow denotes a gene USR, blue denotes a small molecule chemical USR. (**F**) Top upstream regulators (USRs) of the concave vs. non-curve DEGs that are predicted to be either activated or inhibited. Yellow denotes a gene USR, blue demotes a small molecule chemical USR. (**G**) Top predicted relevant biological functions of the convex vs. non-curve dataset and (**H**) of the concave vs. non-curve dataset.

Next, we used ingenuity pathway analysis (IPA, Qiagen, UK) software to identify the dysregulated canonical signalling pathways related to each of the two DEG datasets i.e., convex curve vs. non-curve, and concave curve vs. non-curve. The most significant pathway identified for each comparison was different; the convex vs. non-curve comparison revealed eukaryotic initiation factor 2 (EIF2) signalling as the most dysregulated pathway (-log(p-value) 11.7), (Fig. 2A), with the network summary (Fig. 2B) highlighting dysregulated cell division and proliferation including activation of DNA promoters, transcription of RNA and entry into cell cycle phases. In contrast, the concave vs. non-curve comparison revealed integrin linked kinase (ILK) signalling as the most dysregulated pathway (-log(p-value) 7), (Fig. 2C), with the network summary (Fig. 2D) predominantly highlighting processes of bone and osteoblast differentiation, and fibrogenesis. These findings suggest that convex and concave osteoblasts may be influenced by distinct biological pressures, potentially reflecting differences in mechanical loading or microenvironmental cues across the curve apex. Notably, pathway analysis revealed that both comparisons (i.e. concave vs. non-curve; convex vs. non-curve) shared several dysregulated pathways, namely, pro-inflammatory cytokine signalling pathways including IL-6, IL-8 and IL-17 A. This convergence suggests a common inflammatory component within the curve apex that may act alongside region-specific signalling changes in AIS pathophysiology.

To further interrogate potential drivers of these transcriptional changes, we performed upstream regulator analysis to identify genes and drugs whose predicted activity states could explain the observed DEG patterns. Upstream regulator analysis of the convex vs. non-curve DEGs predicted the dimer PDGF-BB (composed of two Platelet-derived growth factor subunit B) to be the most inhibited, with the growth factors EGF and HGF and transcriptional regulators (CREB1, RELA, LARP1) also predicted to be significant inhibited upstream regulators (Fig. 2E). Of the predicted activated upstream regulators, the PI3K inhibitor LY294002 was predicted to be the most activated, with other chemical inhibitors (U0126, PD98059) and transcription regulators (MLXIPL, MYCN, ZFP36) also highlighted as significant activators (Fig. 2E). In contrast, upstream regulator analysis of the concave vs. non-curve comparison (Fig. 2F), revealed TGFB1 as the most highly inhibited regulator, with the TGF-β family as a whole, and the transcription regulator SMAD3 also predicted to be significant inhibited regulators. Of the predicted activated upstream regulators identified, the JNK inhibitor SP600125 was the most highly activated, with LY294002 again predicted along with microRNAs (miR-30c-5p, miR-338-3p, miR-29b-3p) to be significant activated upstream regulators (Fig. 2F). Finally, predicted biological functions relevant to bone were filtered from the convex vs. non-curve comparison, with most predicted to be downregulated by Z-Score, with the most significant by p-value being growth of connective tissue (Fig. 2G). For the concave vs. non-curve comparison, most functions were again predicted to be downregulated with differentiation of connective tissue cells being the most significant by p-value (Fig. 2H). These results highlight region-specific regulatory and upstream mechanisms that are distinct to each curve site. These results indicate that distinct upstream regulatory networks operate at each side of the curve apex, and with the identification of multiple chemical inhibitors as upstream regulators, prompted us to functionally test whether pharmacological modulation could alter osteoblast behaviour.

### he mTOR pathway inhibitor rapamycin alters osteogenic functional activity in a human osteoblast cell line

**Fig. 3:**
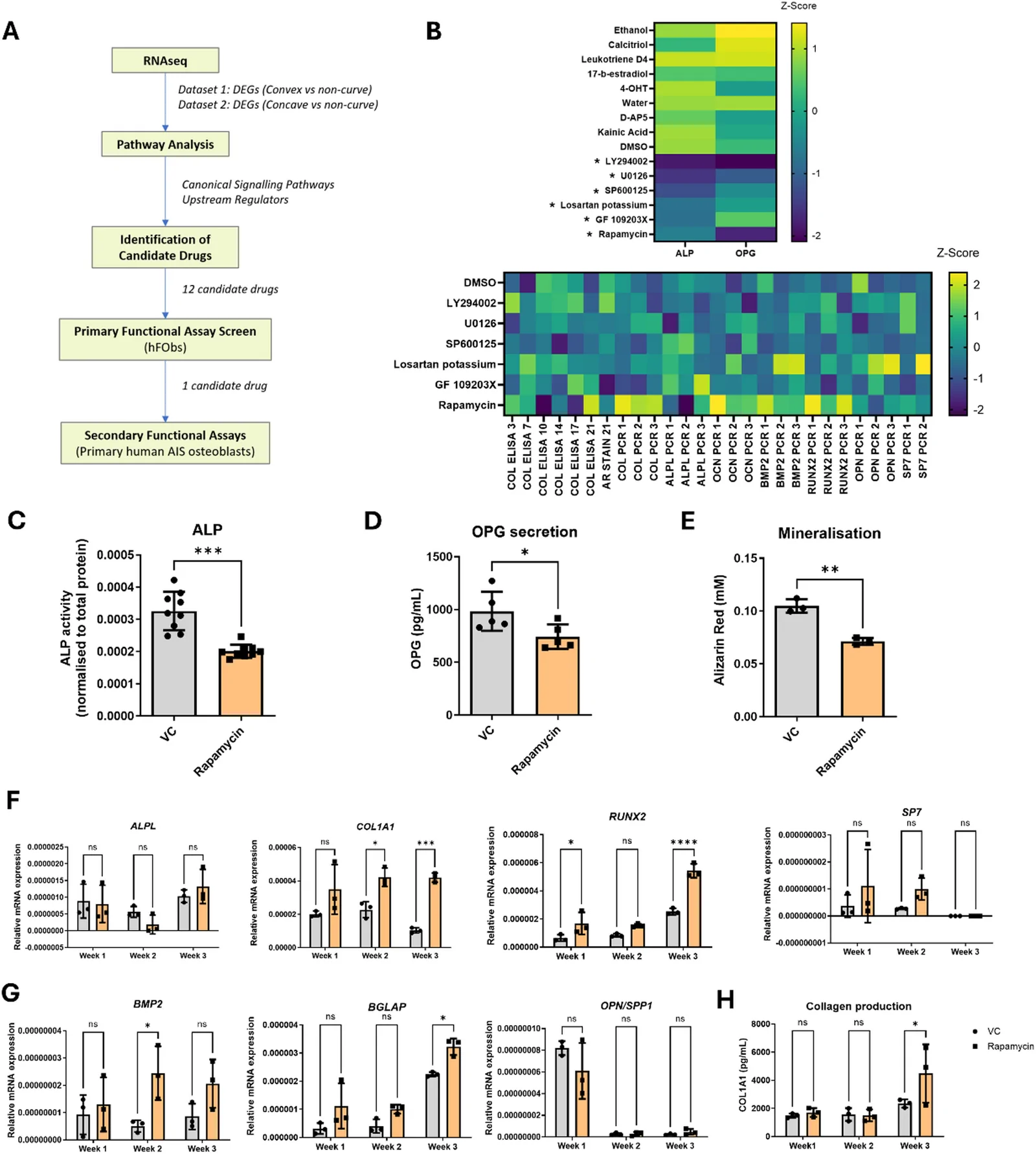
Rapamycin is a functional modulator of human osteoblasts. (**A**) Workflow schematic of the approach for the identification and screening of Upstream regulators (USRs), with functional testing in the human hFOB osteoblast cells and then in primary AIS patient osteoblasts. (**B**) Summary heat maps (by Z-score) of primary functional screening assays of USR for short term (top) and long term (bottom) functional outputs. (**C**) Effects of short-term (24 h) rapamycin treatment (1 nM) on Alkaline phosphatase activity and (**D**) Osteoprotegerin secretion in hFOB cells, compared to vehicle control (DMSO). (**E**) Effect of long-term (21 day) rapamycin treatment on mineralisation ability in hFOB cells compared to vehicle control (DMSO), assessed by Alizarin Red. (**F**) Gene expression changes following rapamycin treatment of hFOB cells for osteoblast differentiation markers and (**G**) mineralisation initiation markers after 1-, 2- and 3-weeks of treatment. (**H**) Effects of rapamycin treatment on collagen production in hFOB cells over 3 weeks compared to vehicle control. Statistical significance was determined by two-tailed T-test (C, D, E) or 2-way ANOVA with Bonferroni’s correction (F, G, H), where *p* < 0.05=*, *p* < 0.01=** and *p* < 0.001=***.

In order to identify and test candidate drugs that could modulate the phenotype of AIS osteoblasts at the curve apex, we filtered the upstream regulators identified from pathway analysis to include only commercially available small molecule inhibitors/activators. This provided a list of 12 candidate drugs (Supplementary Table S2), which were screened to assess acute (24 h) and chronic (21 day) effects on multiple parameters of osteoblast function (including mineralisation, alkaline phosphatase activity (ALP), osteoprotegerin (OPG) secretion and gene expression of modulators of osteoblast function) using the human foetal osteoblast cell line hFOb 1.19 (Fig. 3A). For all screen parameters, 30,000 cells were seeded per well, then cultured in osteogenic media for 3 days before treatment given, also in osteogenic media. Across these multiple parameters of osteoblast function, the mTOR inhibitor rapamycin was found to elicit the most pronounced and consistent effects, as summarised by the heatmap (Fig. 3B; Supplementary Fig. 1–5). Acute (24 h) treatment with rapamycin (1 nM) significantly reduced both ALP enzymatic activity (Fig. 3C) and OPG secretion (Fig. 3D) compared to vehicle control. Furthermore, chronic treatment with rapamycin showed a significant reduction in the ability of the osteoblasts to form and deposit mineral in their matrix, assessed by alizarin red quantification (Fig. 3E).

To investigate whether these functional changes are a result of rapamycin differentially affecting key genes associated with osteoblast differentiation and maturation, cells were dosed (1 nM) every three days for 21 days and RNA extracted after 7-, 14- and 21-day timepoints. For osteogenic markers (Fig. 3F), 7 days of rapamycin treatment resulted in no significant effect on *ALPL* (tissue non-specific alkaline phosphatase) expression, a significant increase in *RUNX2* (runt-related transcription factor 2) expression, which was also increased at 21 days and a significant increase in *COL1A1* (alpha-1 type I collagen) expression at 14 and 21 days. *SP7* (osterix) expression was highly variable, and statistically significant differences were not detected between rapamycin treatment and vehicle control at any timepoint. Notably, by day 21, *SP7* expression was undetectable in either rapamycin treated or control cells.

For markers associated with mineralisation initiation (Fig. 3G), *BMP2* (bone morphogenetic protein 2) was significantly higher at 14 days of rapamycin treatment compared to vehicle control, whereas *SPP1* (osteopontin) showed no significant differences between rapamycin or control cells at any time point, and expression was markedly reduced from day 7 onwards in both control and rapamycin treated cells. Secreted type-1 collagen (COL1A1), also a key functional indicator of osteoblast activity, was also significantly increased at week three with rapamycin treatment (Fig. 3H) compared to vehicle control. These results collectively suggest that rapamycin is capable of modulating both the function and differentiation of osteoblasts, prompting further investigation of mTOR signalling in primary AIS osteoblasts.

### mTOR signalling dysregulation in curve apex osteoblasts and its association with curve severity

**Fig. 4:**
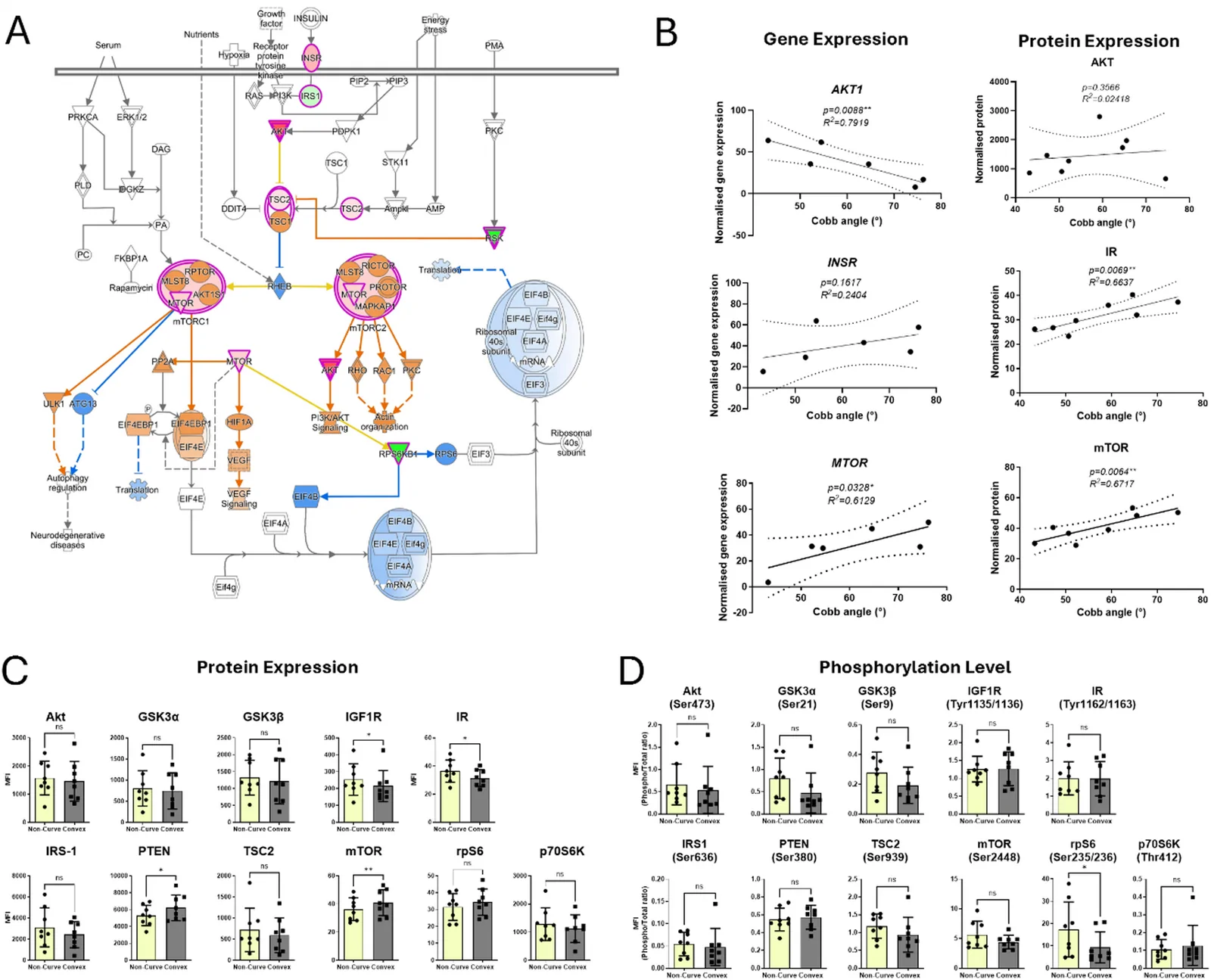
Dysregulated mTOR signalling in curve apex convex osteoblasts correlates with curve severity. (**A**) Overlaid gene expression changes in the mTOR pathway from bulk RNA-seq using IPA (Qiagen); orange = predicted activation, blue = predicted inhibition. (**B**) Correlation between the expression of mTOR signalling in convex curve apex cells and curve severity. Gene (*n* = 6) and protein (*n* = 8) expression of mTOR pathway components AKT1/AKT, INSR/IR and MTOR/mTOR were measured in convex cells by qPCR and Western Blot respectively. Curve severity was measured by Cobb angle (°) (95% confidence bands shown). (**C**) Protein expression of mTOR signalling components, including mTOR, between matched non-curve and convex AIS spinal osteoblasts. (**D**) Phosphorylated protein expression of mTOR signalling components, including mTOR, between matched non-curve and Convex AIS spinal osteoblasts. Statistical significance was determined by Pearson’s correlation following linear regression (B) or by paired T-test (C, D) where *p* < 0.05=*, *p* < 0.01=** and *p* < 0.001=***.

Given our finding that rapamycin elicits functional responses in osteoblasts, we next mapped the transcriptomic dataset from the convex v non-curve comparison onto the mTOR signalling pathway to interrogate which pathway components were dysregulated (Fig. 4A). Much of the pathway was predicted to be activated, including both mTOR complexes (mTORC1 and 2), although translation subunits were predicted to be inhibited (Fig. 4A). Bolstering the connection between dysregulated mTOR signalling in osteoblasts at the curve apex and AIS pathology, we also found that mTOR pathway components correlated significantly with curve severity at the convex site (Fig. 4B). AKT1 gene expression negatively correlated with curve severity, whilst IR protein expression, and mTOR gene and protein expression positively correlated with curve severity.

Finally, we profiled mTOR pathway protein (Fig. 4C) and phosphorylation (Fig. 4D) levels, both upstream and downstream of mTOR itself, in patient matched convex and non-curve osteoblasts. For markers upstream of mTOR, we found significant decreases in insulin receptor levels (IGF1R and IR) in osteoblasts isolated from convex regions compared to patient-matched non-curve cells, as well as a significant increase in PTEN expression. mTOR itself was found to be expressed significantly higher in the convex cells, with no difference in downstream pathway component expression. Interestingly, no upstream markers of mTOR, nor mTOR itself, were found to be significantly different at the phosphorylation level between convex and non-curve cells. For markers downstream of mTOR, RPS6 had significantly lower phosphorylation levels in the convex cells. Together, these findings suggest that osteoblasts at the curve apex show a dysregulated mTOR signalling state characterised by altered signal integration and reduced translational output rather than canonical mTOR activation.

### The mTOR inhibitor rapamycin modulates the function and phenotype of AIS spinal osteoblasts

**Fig. 5:**
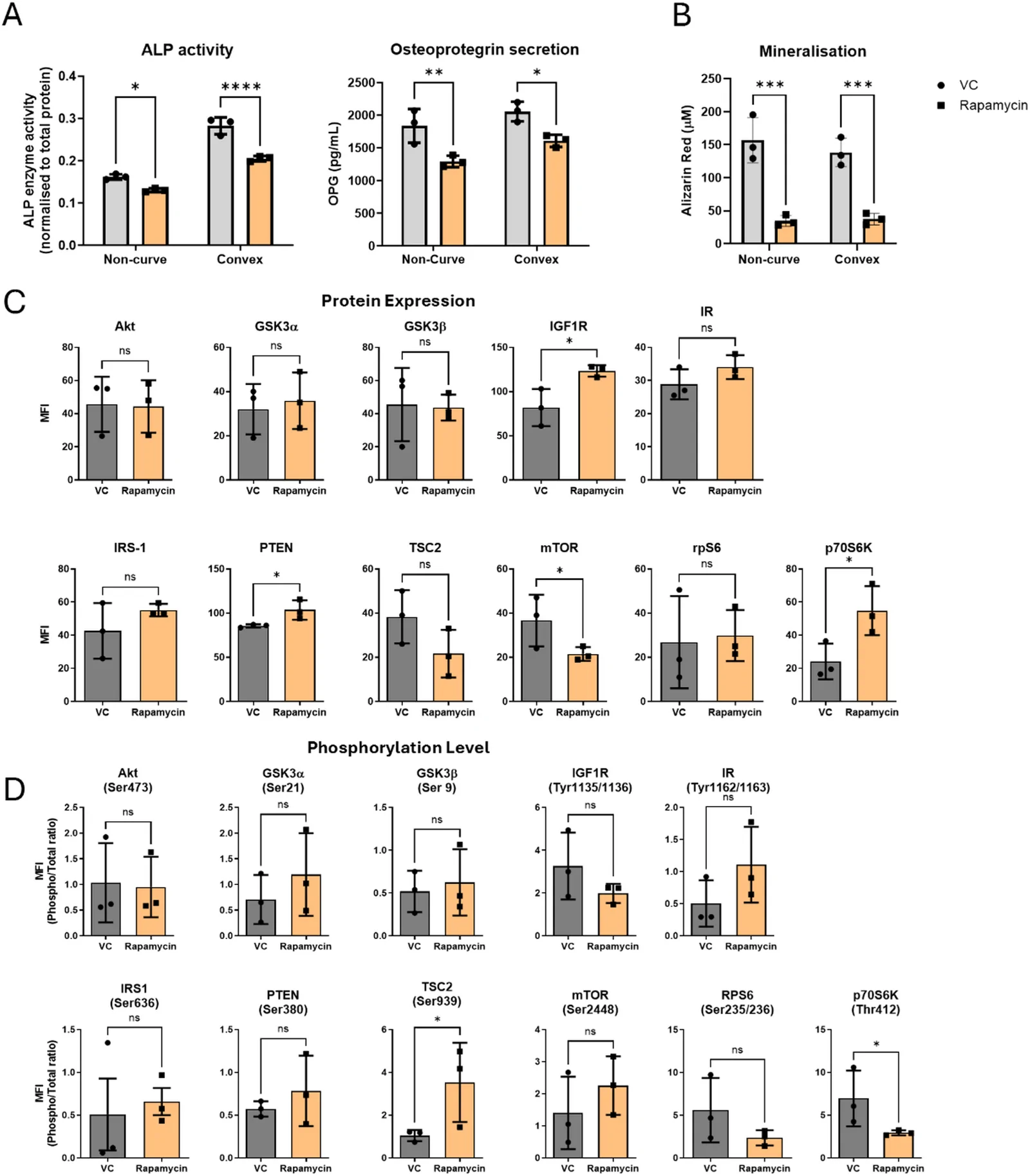
Rapamycin modulates the function and phenotype of AIS spinal osteoblasts. (**A**) Effects of short-term rapamycin treatment on alkaline phosphatase activity and osteoprotegerin secretion in matched convex and non-curve osteoblasts. (**B**) Effects of long-term rapamycin treatment on mineralisation in matched convex and non-curve osteoblasts. (**C**) Effects of rapamycin on total protein and (**D**) protein phosphorylation levels in convex osteoblasts, compared to vehicle control DMSO (VC). Statistical significance was determined 2-way ANOVA with Bonferroni’s correction (A-B), or by two-tailed T-test (C-D) where *p* < 0.05=*, *p* < 0.01=** and *p* < 0.001=***.

Next, the ability of rapamycin to modulate the phenotype of primary osteoblasts from the curve apex (convex) and from outside the curve (non-curve) in AIS patients was assessed. We examined the functional effect of rapamycin treatment on the cells from the different spinal sites. Similar to that observed using the hFOb cell line, short term treatment of rapamycin (1 nM) significantly reduced ALP activity in primary AIS patient osteoblasts compared to vehicle control (Fig. 5A). Notably, the greatest effect was observed in convex curve osteoblasts (average 27% reduction) compared to an average 19% reduction in non-curve cells. OPG secretion was also reduced in convex cells with treatment (average 21.5% reduction) but had the greatest effect in non-curve cells (average 29% reduction). Mineralisation of both convex and non-curve cells was reduced at similar rate after chronic treatment (Fig. 5B).

Next, we profiled the effect of rapamycin on the expression and their phosphorylation status of other mediators within the mTOR pathway on convex osteoblasts. Rapamycin significantly increased IGF1R, PTEN and p70S6K protein expression in convex cells (Fig. 5C). Conversely, rapamycin significantly reduced expression of mTOR itself. Additionally, rapamycin increased phosphorylation of TSC2, whilst decreasing phosphorylation of p70S6K (Fig. 5D).

## Discussion

Dysregulated bone remodelling is a driving factor in numerous musculoskeletal diseases across the lifespan. Here, we have demonstrated for the first time that the mTOR signalling pathway is altered in AIS osteoblasts at the curve apex and provide evidence that pharmacological inhibition of mTOR signalling may, in part, reverse this pathotype. Importantly, our data further demonstrate that these alterations are highly spatially dependent, with osteoblasts from convex and concave regions of the curve apex exhibiting distinct molecular and functional phenotypes compared to non-curve spinal sites. Whilst both regions exhibit dysregulation, our downstream analyses focused on convex osteoblasts due to their more pronounced functional activity and responsiveness to mTOR modulation, suggesting a potentially dominant role in driving asymmetric bone remodelling. Nevertheless, the reduced osteogenic signature observed in concave cells highlights an important complementary pathological mechanism that warrants further investigation.

Patient-matched convex and concave spinal tissue in AIS are known to display different tissue parameters including BMD and trabecular thickness^25^, which supports the concept that different intrinsic pathological drivers are responsible for mediating the osteoblast pathotype at each side of the curve apex. Pathway analysis of differentially expressed gene datasets of spinal osteoblast cells derived from the apex of the curve revealed distinct and shared dysregulated biological pathways between curved (concave and convex) and osteoblasts from non-curve sites. Consistent with this, principal component analysis of osteoblast transcriptomes revealed clustering by spinal site rather than by patient-specific variables such as age or sex, indicating that local microenvironmental cues dominate osteoblast identity in AIS. Pathway analysis of differentially expressed gene datasets of spinal osteoblast cells derived from the apex of the curve revealed distinct and shared dysregulated biological pathways between curved (concave and convex) and osteoblasts from non-curve sites.

In the convex vs. non-curve comparison, EIF2 signalling was the most dysregulated, with enriched pathways related to cell proliferation and division, and PDGF-BB identified as the most inhibited upstream regulator. In contrast, the concave vs. non-curve comparison showed ILK signalling as the most dysregulated, with pathways associated with bone and osteoblast differentiation, and TGFB1 as the most inhibited upstream regulator. These findings suggest that convex osteoblasts may exist in a state of altered translational control and cellular stress, whereas concave osteoblasts display changes more closely aligned with matrix interaction and differentiation processes. Despite these differences, both comparisons shared dysregulation of key pro-inflammatory cytokine signalling pathways, including IL-6, IL-8, and IL-17 A, and featured common upstream regulators such as the PI3K inhibitor LY294002, potentially indicating increased inflammatory responses in spinal tissue at either side of the curve apex. This convergence on inflammatory signalling supports emerging evidence that low-grade inflammation contributes to abnormal bone remodelling in AIS and may act synergistically with mechanical and metabolic stressors. In addition, these region-specific differences may reflect underlying variations in bone microarchitecture, such as altered trabecular organisation and cortical thickness and bone porosity. Convex regions are typically subjected to altered mechanical loading environments that favour increased bone turnover and remodelling, whereas concave regions often exhibit reduced bone formation and altered matrix organisation. These structural differences are likely to influence osteoblast behaviour through mechanotransductive pathways. mTOR signalling is a known integrator of mechanical, metabolic, and nutrient-sensing inputs, and has been implicated in regulating osteoblast proliferation, matrix production, and mineralisation. Dysregulation of mTOR signalling in convex osteoblasts, as observed in this study, may therefore contribute to aberrant matrix deposition and reduced mineralisation efficiency despite elevated osteogenic gene expression, consistent with an uncoupled or maladaptive response. Furthermore, the identification of EIF2 signalling dysregulation suggests a link to cellular stress responses, which may arise from altered mechanical strain or metabolic demand within the curve apex microenvironment. Collectively, these findings support a model in which region-specific differences in bone microarchitecture and mechanical loading interact with dysregulated mTOR signalling to drive asymmetric bone remodelling in AIS.

Screening a panel of identified pharmacological upstream regulators as candidate drugs that could mediate the phenotype of osteoblasts at the curve apex revealed that rapamycin, an mTOR inhibitor, the most effective candidate. The mammalian/mechanistic target of rapamycin (mTOR) is well known as a central regulator of cell growth and metabolism. mTOR is known for roles in bone remodelling^26^ and mTORC1 specifically was shown to facilitate bone healing in a mouse model^27^. The mTOR pathway has also previously been implicated in scoliosis, albeit in animal models, including rat^28^, and zebrafish^29^ systems, underscoring its relevance to spinal development and deformity.

mTOR exists as two complexes: mTORC1 and mTORC2. mTORC1 activation is required for preosteoblast proliferation, but overactivation can impair differentiation and maturation, thought to be through Notch pathway activation^30^. However, when mTORC1 is disrupted, there is decreased matrix synthesis and mineralization, suggesting that it can promote the transition to mature osteoblasts^31^. Interestingly, inhibition of mTOR was found to suppress osteogenic gene expression whilst under strain^32^. Rapamycin selectively inhibits mTORC1, which is responsible for promoting protein synthesis, cell growth and metabolism. These findings collectively highlight the highly context- and stage-dependent role of mTOR signalling in osteoblast biology. Given these complex and sometimes opposing roles, systemic inhibition of mTOR is unlikely to represent a viable therapeutic strategy in adolescents. Instead, future approaches may focus on selective modulation of upstream regulators (e.g., PI3K/AKT signalling), as well as the development of localised or targeted delivery strategies to minimise systemic effects.

Rapamycin has shown promise for treating several bone diseases, including those involving aging or inflammation such as osteoporosis^33^ and osteoarthritis^34^, and those involving abnormal bone remodelling: heterotopic ossification^35^, osteolytic disease^36^. However, the potential of rapamycin as a therapeutic has been evaluated in predominantly older populations. Preclinical studies assessing its skeletal effects have produced mixed results, with reports of reduced bone density following prolonged treatment^37^, contrasted by minimal effects on bone microarchitecture or strength with short term, low dose treatment^38^.

In our study we found that rapamycin suppressed functional human osteoblast markers (ALP, OPG) and mineralization capacity in both a human osteoblast cell line and in AIS patient primary spinal osteoblasts from the curve apex. Furthermore, rapamycin, increased the expression of key osteogenic genes *RUNX2*, *COL1A1*, as well as increased collagen production. The continued higher expression of *RUNX2*, typically considered an early osteoblast marker^39^, suggests that rapamycin may have not only anti-proliferative effects but may also disrupt osteoblast differentiation, consistent with a block in late-stage maturation. Notably, the persistence of *BGLAP* expression and the absence of detectable *SP7* expression at later timepoints suggest that mTOR inhibition may uncouple early differentiation from terminal maturation rather than fully abrogating osteogenesis. The downregulation of *SPP1* at later timepoints may reflect the loss of *SP7*, which along with *RUNX2*, is required for its expression^40–42^. These findings also highlight that osteoblast-mediated bone formation is only one component of overall bone remodelling, and future studies incorporating osteoclast activity and bone resorption markers will be important to fully understand the balance of skeletal turnover in AIS.

Further evidence for the role of dysregulated mTOR signalling in AIS pathogenesis was garnered upon finding that the expression of several mediators of mTOR signalling were not only differentially expressed in curve apex spinal osteoblasts but that their expression correlated with curve severity. However, of note, although mTOR expression was increased in convex curve apex osteoblasts and the pathway appears broadly activated, translation-related outputs (e.g., RPS6 phosphorylation) were reduced, suggesting a dysregulated or uncoupled mTOR response. Specifically, upstream mTOR signalling was dampened, evidenced by reduced insulin receptor expression and increased PTEN, while downstream translational activity was suppressed despite elevated mTOR levels. This decoupling suggests that altered mTOR signalling in AIS osteoblasts is not driven by canonical phosphorylation-based activation but may instead reflect impaired signal integration and translational control. The reduced expression of IGF1R and IR in convex cells may reflect crosstalk with other spinal tissues or systemic metabolic influences and may contribute to altered autophagy regulation rather than increased protein synthesis. This interpretation is supported by the identification of EIF2 signalling as the most dysregulated pathway in the convex vs. non-curve comparison, a pathway closely linked to cellular stress responses and the unfolded protein response (UPR). Together, these features may underpin both the altered functional phenotype of convex osteoblasts and their association with curve severity in AIS. Importantly, while these associations do not establish causality, they suggest that dysregulated mTOR signalling may contribute to disease progression, particularly through sustained asymmetric bone remodelling at the curve apex.

There are several limitations to this study. First, the relatively small patient cohort reflects the challenges of obtaining spinal tissue during corrective surgery in adolescents. As a result, sex-specific analyses were not feasible, despite the known influence of sex hormones on bone metabolism and insulin/mTOR signalling^43^. Second, while we identified strong associations between osteoblast phenotypes, mTOR pathway components and curve severity, causality cannot be inferred. Longitudinal sampling of spinal tissue is not ethically feasible, highlighting the need for future studies to identify circulating biomarkers reflective of curve apex osteoblast phenotypes. Such biomarkers could enable longitudinal monitoring of disease progression and treatment response. Importantly, the absence of non-scoliotic healthy control spinal tissue represents an important limitation, and future work incorporating appropriate control samples where feasible would further strengthen mechanistic interpretation. Additionally, limited tissue availability constrained architectural and mechanistic analyses, and in vitro studies were inherently limited by the proliferative capacity of primary osteoblasts. Patient-specific bone structural parameters, including bone mineral density and microarchitectural measures, were additionally not available for the subset of samples used in this study, limiting our ability to directly correlate molecular findings with bone quality in the same individuals.

In conclusion, we demonstrate that AIS is associated with intrinsic, spatially defined osteoblast dysfunction at the curve apex, characterised by dysregulated mTOR signalling. Rapamycin partially modulated this phenotype, revealing mTOR as a key mechanistic node linking inflammatory, metabolic and stress-related pathways in AIS. While systemic mTOR inhibition is unlikely to be clinically viable in adolescents, our findings provide a strong rationale for exploring targeted modulation of mTOR-related pathways as a strategy to correct asymmetric bone remodelling in AIS and potentially other disorders of abnormal skeletal growth.

## Materials and methods

### Patient recruitment and collection of spinal tissues

Facet spinal tissue was collected perioperatively from AIS patients undergoing surgery at the Royal Orthopaedic Hospital, Birmingham, UK. Prior informed patient (and/or parent/carer) consent was obtained. The study was approved by the West Midlands - South Birmingham Research Ethics Committee (reference 19/WM/0083). The study was conducted in compliance with the Declaration of Helsinki; clinical data are summarised in Table 1.

### Isolation of spinal osteoblasts and cell culture

Facet bone chips were washed three times in Dulbecco’s Modified Eagles Media (DMEM) (Cat: 41965062; Gibco) containing 100 U/mL penicillin streptomycin (Cat: 15140122; Gibco) and cultured in differentiation media (DMEM, 10% FBS, 100 U/mL penicillin streptomycin, 1% non-essential amino acids (NEAA) (Cat: 11140035; Gibco), 2 mM β-glycerophosphate (Cat: G9422; Sigma-Aldrich), 50 µg/mL L-Ascorbic acid (Cat: A5960; Sigma-Aldrich), 10 nM Dexamethasone (Cat: D4902; Sigma-Aldrich)). Media was changed every three days and bone chips removed upon the appearance of adhered osteoblasts. Primary cells were then used between passages 1 and 3.

hFOb 1.19 cells (CRL-3602; ATCC) were cultured in either basal media, composed of DMEM/F-12, no phenol red (Cat:11580546; Gibco) supplemented with 10% FBS and 0.3 mg/mL G418 (Cat: 4727878001; Sigma-Aldrich) or osteogenic media, composed of basal media supplemented with 10^− 8^ M menadione and 100 µg/mL L-Ascorbic acid (all from Sigma-Aldrich). Unless stated otherwise, cells were seeded then cultured in osteogenic media for 3 days before treatment or assay. hFOb cells were used between passages 10 and 14.

###  Transcriptomic analysis

RNA was extracted using a RNeasy Mini Kit (Cat: 74104; Qiagen) according to manufacturer’s instructions. Cells were collected by addition of RLT buffer supplemented with β-mercaptoethanol and incubated on ice for 20 min. RNA was quantified using a NanoDrop One (Thermo Scientific).

For RNA sequencing, RNA integrity (RIN) was evaluated in P1 primary osteoblasts by bioanalyzer (Agilent) and samples with a RIN > 7 were deemed acceptable. Sequencing was performed by Birmingham Genomics using QuantSeq 3’ mRNA-Seq Library Prep Kit FWD for Illumina (Lexogen). Data processing was performed in Qlucore, (Lund, Sweden) and pathway analyses were performed using Ingenuity Pathway Analysis (IPA) (V21.0; Qiagen). Differentially expressed genes (fold change of ± > 1.5, *p* < 0.05) were analysed using the core functional analysis feature to identify significant canonical pathways and cellular processes for each comparison (concave/non-curve, convex/non-curve).

For selective gene expression analysis, relative mRNA expression was determined by Quantitative Real-Time Polymerase Chain Reaction (RT-qPCR) in hFOb cells. This was performed using the iTaq™ Universal SYBR^®^ Green One-Step Kit (Cat: 1725151; BIO-RAD), 5 ng total RNA and primers (Supplementary Table S1) (Merck) on a CFX384 C1000 Touch Thermal Cycler (BIO-RAD). After normalisation to housekeeping gene 18 S, data are presented as relative expression unless stated otherwise.

### Alkaline phosphatase activity assay

Alkaline phosphatase activity was assessed at 24 h post treatment. Cells were lysed by addition of RIPA buffer (1X) (Cat: 20–188; Millipore) supplemented with protease and phosphatase inhibitors (Cat: 4693132001, 4906845001; Roche) and placed on ice for 20 min with agitation. Cells were harvested using a cell scraper, passed through a 21-gauge needle, and then centrifuged at 20,000 x g for 15 min at 4 °C. Total protein concentration was determined using BCA Assay (Cat: 23225; Thermo Scientific). Lysates were then added to room temperature alkaline phosphatase yellow (pNPP) liquid substrate (Cat: P7998, Sigma-Aldrich) at a 1:4 ratio in a 96 well plate. The plate was protected from light and incubated at 37 °C on a shaker for 45 min. The plate was then quantified using a synergy HT plate reader at 405 nm absorbance (with 540 nm as a wavelength control). Data is presented as fold change to vehicle control, after normalisation to total protein unless stated otherwise.

###  Alizarin red staining

Alizarin red staining and quantification was performed using an ARed-Q kit (Cat: 8678; Caltag+Medsystems) according to manufacturer’s protocol. Rapamycin (1 nM, Cat: r0161 ; LKT Laboratories) treatment or vehicle control was dosed every 3 days and the cells left to mineralise for 3–4 weeks. Cells were then fixed in 4% paraformaldehyde (PFA) prior to staining with 40 mM Alizarin red solution (ARS) per well. Dye was then extracted using 10% acetic acid, incubated for 30 min at RT. Cells were then transferred to a 1.5 mL Eppendorf tube and vortexed for 30 s before boiling at 85 °C for 10 min. Tubes were then placed on ice before centrifugation at 20,000 x g for 15 min. 10% ammonium hydroxide was then added to 500 µL of supernatant to neutralise the acid. Standards were prepared according to manufacturer’s instructions and both standards and samples were loaded onto a 96-well opaque-walled transparent bottomed plate and read at 405 nm using a Synergy HT plate reader (Biotek). Data are presented in mM after standard curve quantification.

### Luminex and ELISAs

mTOR pathway signalling was assessed using the Total Akt/mTOR 11-plex (Cat: 48-612MAG; Merck-Millipore) and Phosphoprotein kits (Cat: 48-611MAG; Merck-Millipore) according to manufacturer’s instructions. In brief, 1 mg total protein for each sample was loaded in triplicate and the samples incubated overnight at 4 °C in the dark with agitation. The next day, samples were analysed on a Luminex^®^ 200™ system running settings recommended by the manufacturer. Total protein is presented as median fluorescence intensity (MFI), and phosphoprotein results were normalised to the respective total protein.

For the quantification of OPG (human osteoprotegerin/TNFRSF11B) and COL1A1 (human pro-collagen 1 α1), ELISAs (Cat: DY805 and DY6220-05; R&D Systems) were performed on cell supernatants according to manufacturer’s instructions. Data was analysed using a 4-parameter logistic curve and plotted as concentrations.

### Statistical analysis

All statistical analysis was performed in Graphpad Prism v10.5.0. All data are mean ± standard error of mean (SEM) unless stated otherwise. Normality tests on all data were performed first using both Shapiro-Wilk and Kolmogorov-Smirnov tests, with Shapiro-Wilk being used solely on datasets with too small an n for Kolmogorov-Smirnov. Significance levels were set at *P* < 0.05, with individual figure legends indicating which specific statistical test were used.

## Supplementary Information

Below is the link to the electronic supplementary material.

Supplementary Material 1

Supplementary Material 2

## Data Availability

The datasets generated and analysed during the current study are available in the Gene Expression Omnibus (GEO) database (accession number GSE324067).

## References

[1] Konieczny, M. R., Senyurt, H. & Krauspe, R. Epidemiology of adolescent idiopathic scoliosis. *J. Child. Orthop.* **7** (1), 3–9 (2013). 10.1007/s11832-012-0457-4 PMC3566258 24432052

[2] Weinstein, S. L. et al. *Adolescent idiopathic scoliosis Lancet*, **371**(9623): 1527–1537. (2008). 10.1016/S0140-6736(08)60658-3 18456103

[3] Negrini, S. et al. Actual evidence in the medical approach to adolescents with idiopathic scoliosis. *Eur. J. Phys. Rehabil Med.* **50** (1), 87–92 (2014). 24622050

[4] Ogilvie, J. W. et al. The search for idiopathic scoliosis genes. *Spine (Phila Pa. 1976)*. **31** (6), 679–681 (2006). 10.1097/01.brs.0000202527.25356.90 16540873

[5] Patten, S. A. et al. Functional variants of POC5 identified in patients with idiopathic scoliosis. *J. Clin. Invest.* **125** (3), 1124–1128 (2015). 10.1172/JCI77262 PMC4362221 25642776

[6] Xu, L. et al. Common variant of POC5 is associated with the susceptibility of adolescent idiopathic scoliosis. *Spine (Phila Pa. 1976)*. **43** (12), E683–E688 (2017). 10.1097/BRS.0000000000002490 29189569

[7] Hassan, A. et al. Adolescent idiopathic scoliosis associated POC5 mutation impairs cell cycle, cilia length and centrosome protein interactions. 14(3). (2019). 10.1371/journal.pone.0213269 PMC6405090 30845169

[8] Mathieu, H. et al. *Prevalence of POC5 Coding Variants in French-Canadian and British AIS Cohort.* Genes. 12 (7) 1032 (2021). 10.3390/genes12071032 PMC8306370 34356048

[9] Mathieu, H. et al. Genetic variant of TTLL11 gene and subsequent ciliary defects are associated with idiopathic scoliosis in a 5-generation UK family. Sci. Rep., 11. (2021). 10.1038/s41598-021-90155-0 PMC8155187 34040021

[10] Kamtsiuris, P. et al. Prevalence of somatic diseases in German children and adolescents. Results of the German Health Interview and Examination Survey for Children and Adolescents (KiGGS) Bundesgesundheitsblatt Gesundheitsforschung Gesundheitsschutz. *Bundesgesundheitsblatt Gesundheitsforschung Gesundheitsschutz*. **50** (5–6), 686–700 (2007). 10.1007/s00103-007-0230-x 17514453

[11] Daruwalla, J. S. et al. Idiopathic scoliosis. Prevalence and ethnic distribution in Singapore schoolchildren. *J. Bone Joint Surg. Br.* **67** (2), 182–184 (1985). 10.1302/0301-620X.67B2.3980521 3980521

[12] Cilli, K. et al. School screening for scoliosis in Sivas, Turkey. *Acta Orthop. Traumatol. Turc.* **43** (5), 426–430 (2009). 10.3944/AOTT.2009.426 19881324

[13] Nery, L. S. et al. Prevalence of scoliosis among school students in a town in southern Brazil. *Sao Paulo Med. J.* **128** (2), 69–73 (2010). 10.1590/S1516-31802010000200005 PMC10938972 20676572

[14] Soucacos, P. N. et al. School-screening for scoliosis. A prospective epidemiological study in northwestern and central Greece. *J. Bone Joint Surg. Am.* **79** (10), 1498–1503 (1997). 10.2106/00004623-199710000-00006 9378735

[15] Raggio, C. L. Sexual dimorphism in adolescent idiopathic scoliosis. *Orthop. Clin. North. Am.* **37** (4), 555–558 (2006). 10.1016/j.ocl.2006.09.010 17141012

[16] Luk, K. D. et al. Clinical effectiveness of school screening for adolescent idiopathic scoliosis: a large population-based retrospective cohort study. *Spine (Phila Pa. 1976)*. **35** (17), 1607–1614 (2010). 10.1097/BRS.0b013e3181c7cb8c 20453727

[17] Ueno, M. et al. A 5-year epidemiological study on the prevalence rate of idiopathic scoliosis in Tokyo: school screening of more than 250,000 children. *J. Orthop. Sci.* **16** (1), 1–6 (2011). 10.1007/s00776-010-0009-z 21293892

[18] Richards, B. S. et al. Treatment of adolescent idiopathic scoliosis using Texas Scottish Rite Hospital instrumentation. *Spine (Phila Pa. 1976)*. **19** (14), 1598–1605 (1994). 10.1097/00007632-199407001-00008 7939996

[19] Kulis, A. et al. Participation of sex hormones in multifactorial pathogenesis of adolescent idiopathic scoliosis. *Int. Orthop.* **39**, 1227–1236 (2015). 10.1007/s00264-015-2742-6 25804208

[20] Nishida, M. et al. Persistent low bone mineral density in adolescent idiopathic scoliosis: A longitudinal study. *J. Orthop. Sci.* **28** (5), 1099–1104 (2023). 10.1016/j.jos.2022.07.005 35985936

[21] Shao, Z. et al. A targeted antibody-based array reveals a serum protein signature as biomarker for adolescent idiopathic scoliosis patients. *BMC Genom.* **24** (1), 522 (2023). 10.1186/s12864-023-09624-7 PMC10478410 37667193

[22] Sheng, K. et al. The TLR-M-CSF axis is implicated in increased bone turnover and curve progression in adolescent idiopathic scoliosis 27 (Arthritis Research & Therapy, 2025). 10.1186/s13075-025-03535-6 PMC11956469 40165259

[23] Oliazadeh, N., Elbakry, G. K. & Moreau, M. A., Altered mechanotransduction in adolescent idiopathic scoliosis osteoblasts: an exploratory in vitro study*.**Sci. Rep.*, **12**(1). (2022). 10.1038/s41598-022-05918-0 PMC8813918 35115632

[24] He, S. et al. *H., *Phosphorylated heat shock protein 27 improves the bone formation ability of osteoblasts and bone marrow stem cells from patients with adolescent idiopathic scoliosis (JOR Spine, 2023). 10.1002/jsp2.1256 PMC10540826 37780830

[25] Pearson, M. J. et al. Evidence of Intrinsic Impairment of Osteoblast Phenotype at the Curve Apex in Girls With Adolescent Idiopathic Scoliosis. *Spine Deform*. **7** (4), 533–542 (2019). 10.1016/j.jspd.2018.11.016 31202368

[26] Chen, J. & Long, F. mTOR signaling in skeletal development and disease. *Bone Res.* **6**, 1 (2018). 10.1038/s41413-017-0004-5 PMC5802487 29423330

[27] Li, D. et al. Dynamic control of mTORC1 facilitates bone healing in mice. *Bone* **190**, 117285 (2025). 10.1016/j.bone.2024.117285 39426581

[28] Wang, Y., Yi, X. D. & Li, C. D. Suppression of mTOR signaling pathway promotes bone marrow mesenchymal stem cells differentiation into osteoblast in degenerative scoliosis: in vivo and in vitro. *Mol. Biol. Rep.* **44** (1), 129–137 (2017). 10.1007/s11033-016-4089-5 27888418

[29] Sun, X. et al. Dstyk mutation leads to congenital scoliosis-like vertebral malformations in zebrafish via dysregulated mTORC1/TFEB pathway. *Nat. Commun.* **11** (1), 479 (2020). 10.1038/s41467-019-14169-z PMC6981171 31980602

[30] Huang, B. et al. mTORC1 Prevents Preosteoblast Differentiation through the Notch Signaling Pathway. *PLoS Genet.* **11** (8), e1005426 (2015). 10.1371/journal.pgen.1005426 PMC4524707 26241748

[31] Chen, J. & Long, F. mTORC1 Signaling Promotes Osteoblast Differentiation from Preosteoblasts. *PLoS One*. **10** (6), e0130627 (2015). 10.1371/journal.pone.0130627 PMC4474698 26090674

[32] Wang, D. et al. The interactions between mTOR and NF-kappaB: A novel mechanism mediating mechanical stretch-stimulated osteoblast differentiation*. J. Cell. Physiol.*, (2020). 10.1002/jcp.30184 33289098

[33] Luo, D. et al. Rapamycin reduces severity of senile osteoporosis by activating osteocyte autophagy. *Osteoporos. Int.* **27** (3), 1093–1101 (2016). 10.1007/s00198-015-3325-5 26395886

[34] Dhanabalan, K. M. et al. Intra-articular injection of rapamycin microparticles prevent senescence and effectively treat osteoarthritis. *Bioeng. Transl Med.* **8** (1), e10298 (2023). 10.1002/btm2.10298 PMC9842044 36684078

[35] Hu, Y. & Wang, Z. Rapamycin prevents heterotopic ossification by inhibiting the mTOR pathway and oxidative stress. *Biochem. Biophys. Res. Commun.* **573**, 171–178 (2021). 10.1016/j.bbrc.2021.07.060 34419763

[36] Hussein, O. et al. Rapamycin inhibits osteolysis and improves survival in a model of experimental bone metastases. *Cancer Lett.* **314** (2), 176–184 (2012). 10.1016/j.canlet.2011.09.026 22014409

[37] Martin, S. A. et al. Rapamycin impairs bone accrual in young adult mice independent of Nrf2. *Exp. Gerontol.* **154**, 111516 (2021). 10.1016/j.exger.2021.111516 34389472

[38] Devine, C. C. et al. Rapamycin does not alter bone microarchitecture or material properties quality in young-adult and aged female C57BL/6 mice. *JBMR Plus*. **8** (2), ziae001 (2024). 10.1093/jbmrpl/ziae001 PMC10945714 38505525

[39] Komori, T. Regulation of osteoblast differentiation by Runx2. *Adv. Exp. Med. Biol.* **658**, 43–49 (2010). 10.1007/978-1-4419-1050-9_5 19950014

[40] Tu, Q., Valverde, P. & Chen, J. Osterix enhances proliferation and osteogenic potential of bone marrow stromal cells. *Biochem. Biophys. Res. Commun.* **341** (4), 1257–1265 (2006). 10.1016/j.bbrc.2006.01.092 PMC2831616 16466699

[41] Kim, Y. J. et al. The bone-related Zn finger transcription factor Osterix promotes proliferation of mesenchymal cells. *Gene* **366** (1), 145–151 (2006). 10.1016/j.gene.2005.08.021 16314050

[42] Cao, Z. et al. Osterix controls cementoblast differentiation through downregulation of Wnt-signaling via enhancing DKK1 expression. *Int. J. Biol. Sci.* **11** (3), 335–344 (2015). 10.7150/ijbs.10874 PMC4323373 25678852

[43] Khosla, S., Oursler, M. J. & Monroe, D. G. Estrogen and the skeleton. *Trends Endocrinol. Metabolism*. **23** (11), 576–581 (2012). 10.1016/j.tem.2012.03.008 PMC3424385 22595550

