# Supplementary material for: mTOR signalling mediates the spinal osteoblast pathotype at the curve apex in adolescent idiopathic scoliosis
Source: Sci Rep. 2026 Jul 1;16:30300. doi: 10.1038/s41598-026-56019-1 (PMC13623629; doi:10.1038/s41598-026-56019-1)
Supplement: Supplementary file 1 [file 41598_2026_56019_MOESM1_ESM.pdf]

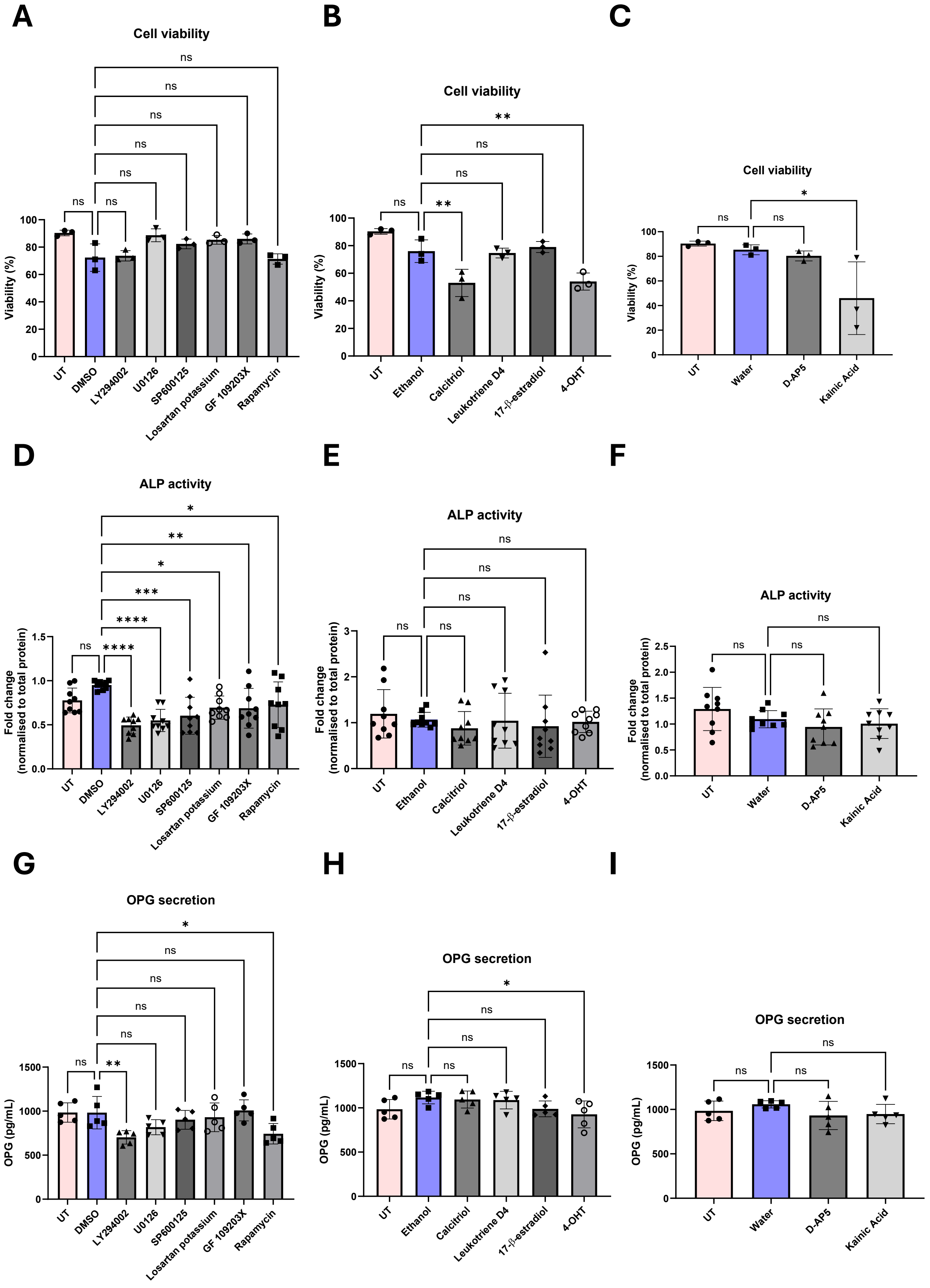

Supplementary Figure 1. Stage 1 functional outputs of high content screen. (A) Effects on cell viability for compounds whose vehicle control is DMSO, (B) ethanol and (C) water after 24 hours treatment (n = 3). (D) Effects on ALP activity for compounds whose vehicle control is DMSO, (E) ethanol and (F) water after 24 hours treatment (n = 9). (G) Effects on OPG secretion for compounds whose vehicle control is DMSO, (H) ethanol and (I) water after 24 hours treatment (n = 5). Statistics: (A - I) were assessed by One-way ANOVA with Dunnett's multiple comparisons post test where P<0.05 = *, P<0.01 = **, P<0.001 = ***, P<0.0001 = ****.

A

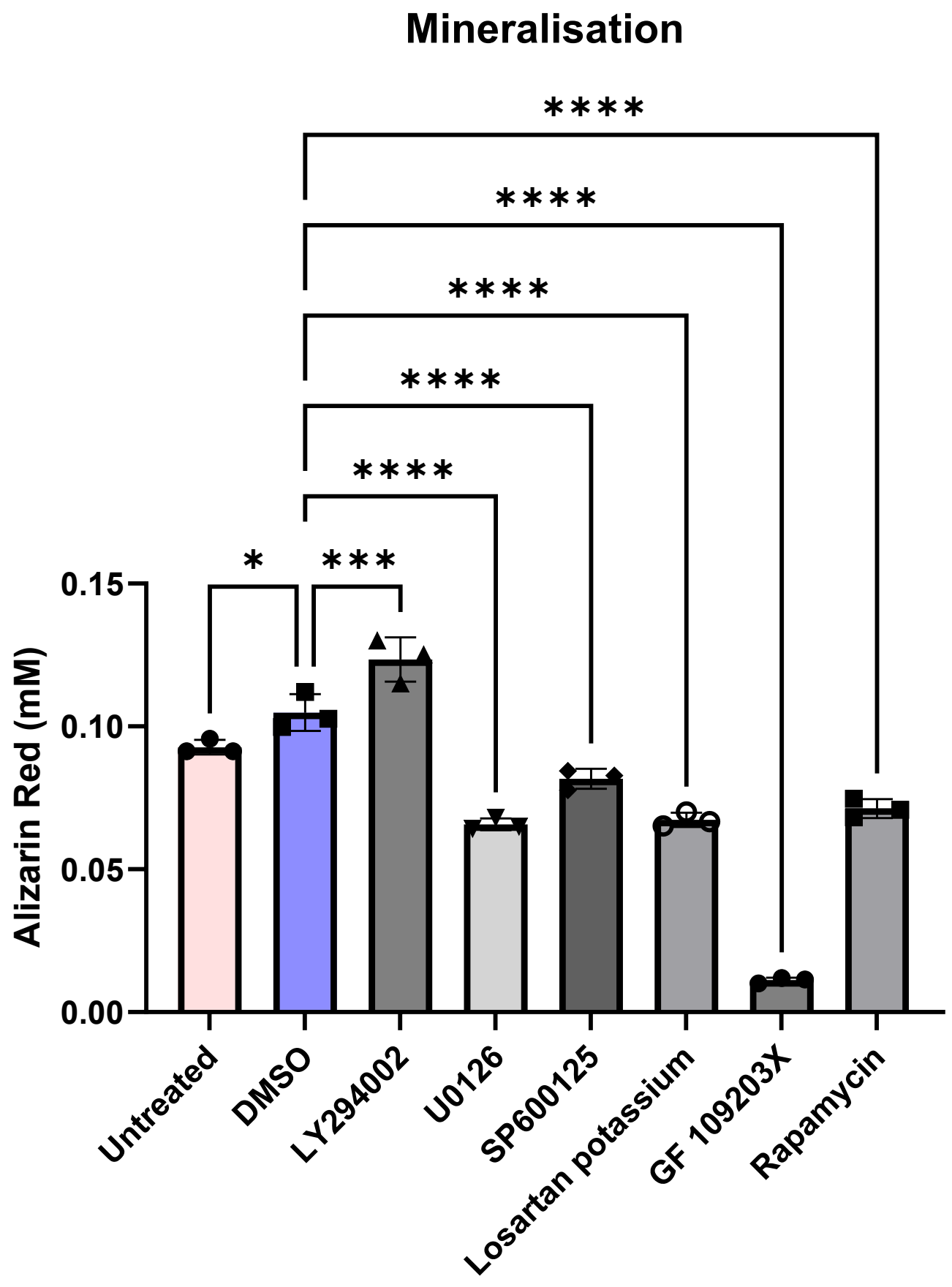

Supplementary Figure 2. Stage 2 functional outputs of high content screen: mineralisation. (A) Effects of compounds on mineralisation ability after 21 days of treatment (n=3). Statistics: (A) was assessed by One-way ANOVA with Dunnett's multiple comparisons post test where P<0.05 = *, P<0.01 = **, P<0.001 = ***, P<0.0001 = ****.

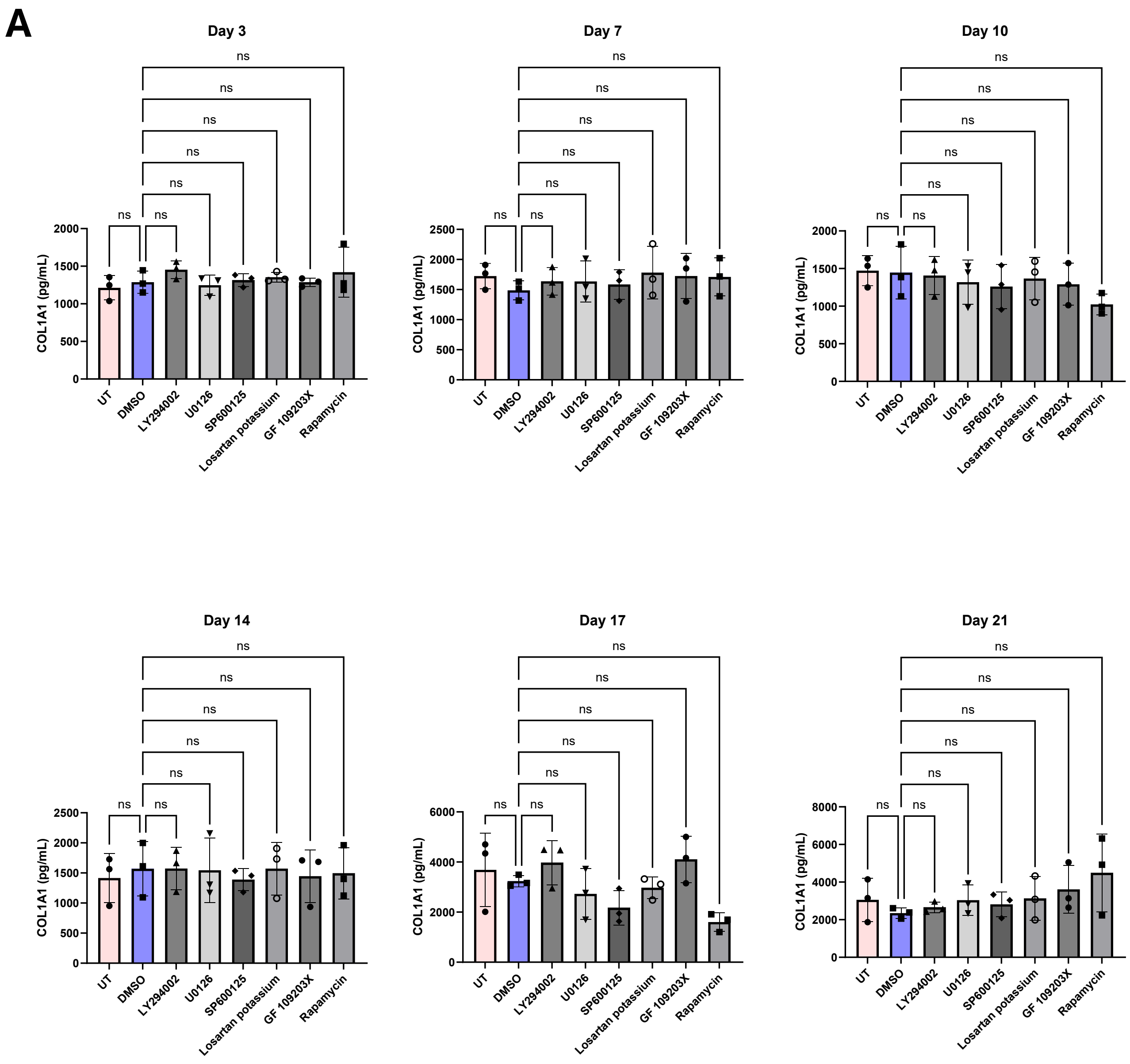

Supplementary Figure 3. Stage 2 functional outputs of high content screen: collagen secretion. (A) Effects of compounds on COL1A1 secretion from day 3 to day 21 of treatment (n=3). Statistics: (A) was assessed by One-way ANOVA with Dunnett's multiple comparisons post test where P<0.05 = *, P<0.01 = **, P<0.001 = ***, P<0.0001 = ****.

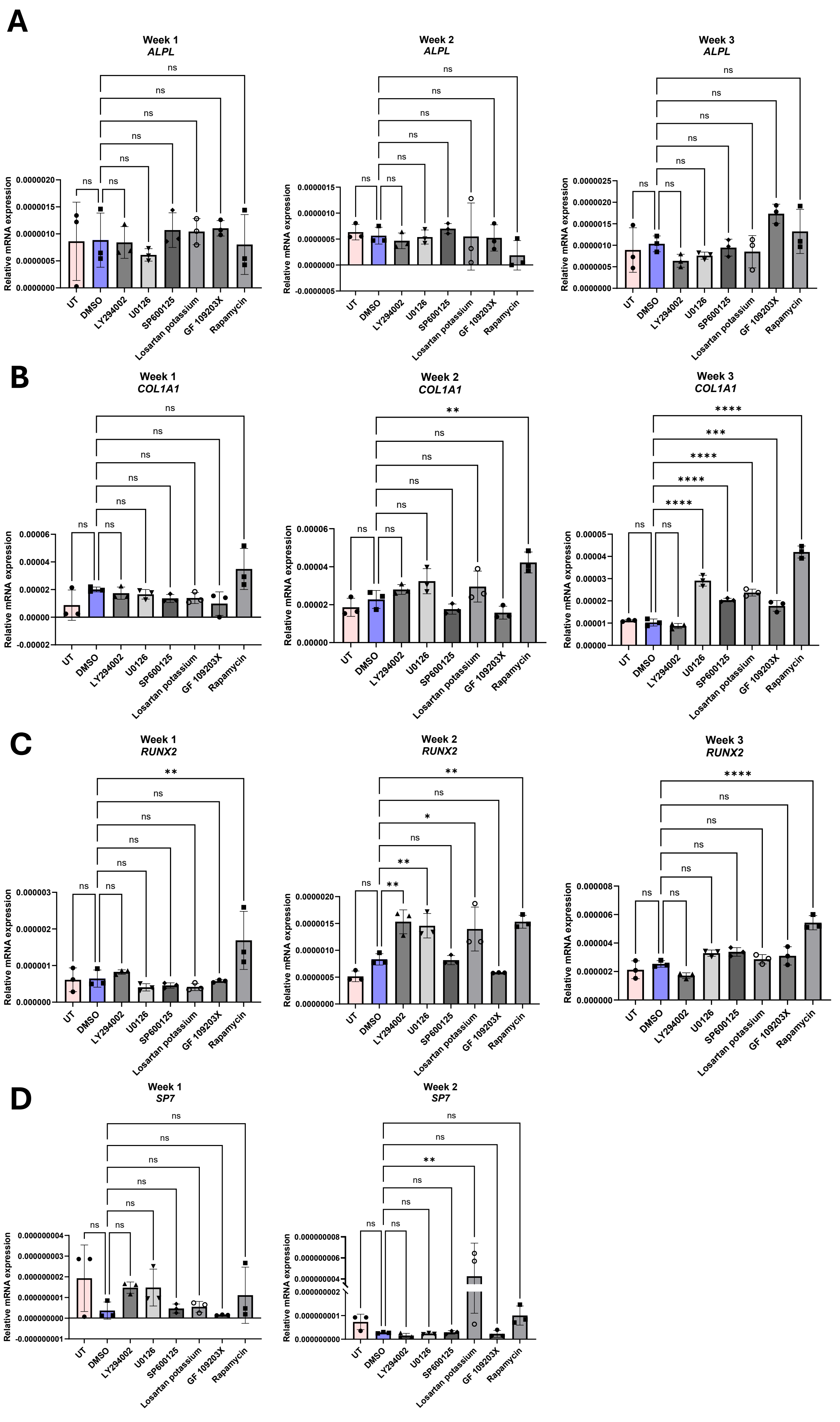

Supplementary Figure 4. Stage 2 functional outputs of high content screen: osteoblast differentiation. (A) Effects of compounds on gene expression of ALPL, (B) COL1A1, (C) RUNX2 and (D) SP7 at weeks 1, 2 and 3 of treatment (n=3).Data is presented as relative expression after normalisation to housekeeping gene 18S. Statistics: (A-D) were assessed by One-way ANOVA with Dunnett's multiple comparisons post test where P<0.05 = *, P<0.01 = **, P<0.001 = ***, P<0.0001 = ****.

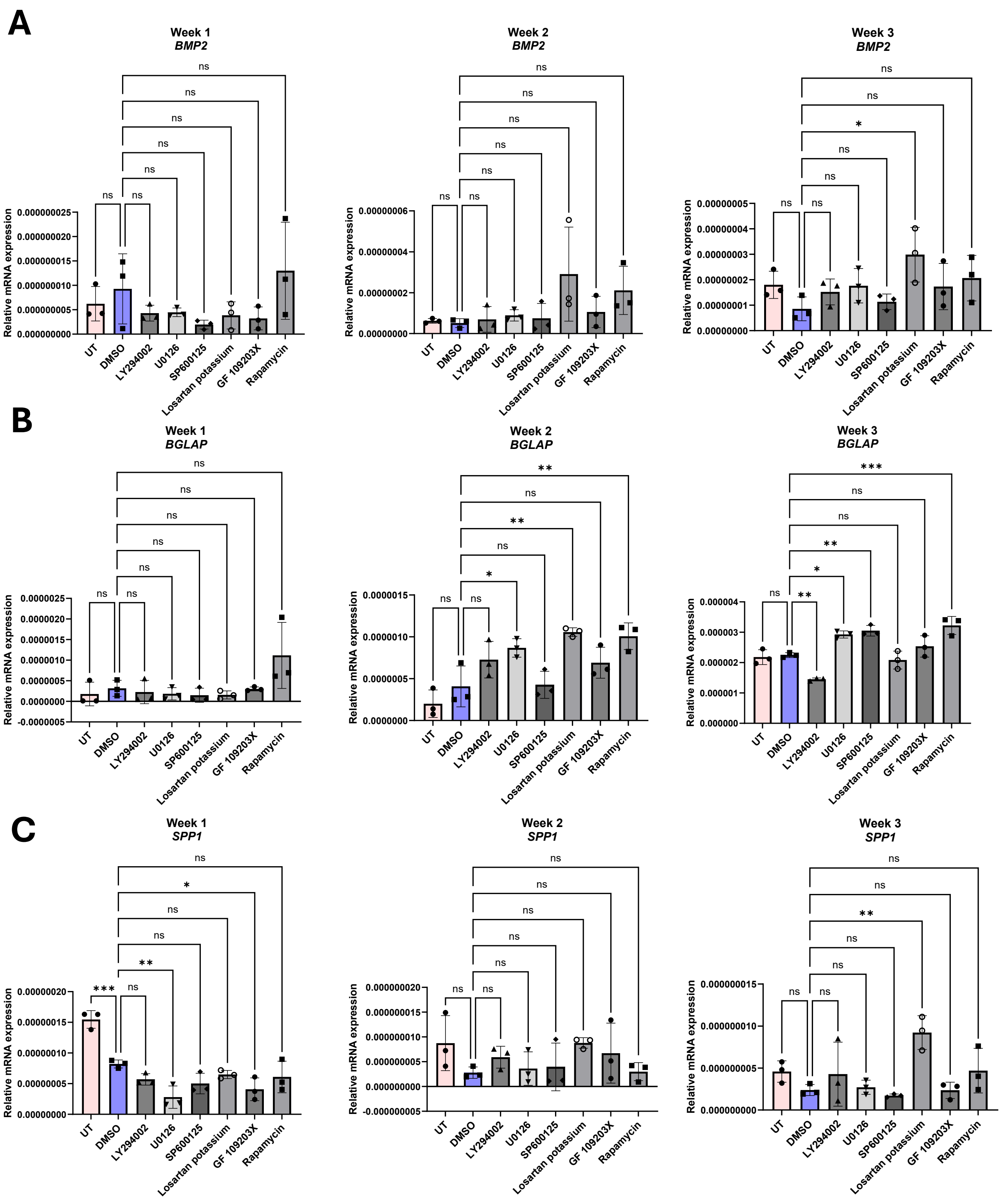

Supplementary Figure 5. Stage 2 functional outputs of high content screen: mineralisation initiation. (A) Effects of compounds on gene expression of BMP2, (B) BGLAP and (C) SPP1 at weeks 1, 2 and 3 of treatment (n=3). Data is presented as relative expression after normalisation to housekeeping gene 18S. Statistics: (A) was assessed by One-way ANOVA with Dunnett's multiple comparisons post test where P<0.05 = *, P<0.01 = **, P<0.001 = ***, P<0.0001 = ****.
