# Supplementary material for: mTOR signalling mediates the spinal osteoblast pathotype at the curve apex in adolescent idiopathic scoliosis
Source: Sci Rep. 2026 Jul 1;16:30300. doi: 10.1038/s41598-026-56019-1 (PMC13623629; doi:10.1038/s41598-026-56019-1)
Supplement: Supplementary file 2 [file 41598_2026_56019_MOESM2_ESM.pdf]

Supplementary Table 1. Primers used for RT-qPCR.

Gene	Name	Forward	Reverse
ALPL	Tissue-nonspecific alkaline phosphatase	ACCCCAAAGGCTTCTTCTTG	TTGGCTTTTCCTTCATGGTG
BMP2	Bone morphogenic protein 2	CAGACCACCGGTTGGAGAGG	CCGGGTTGTTTTCCCACTCG
BGLAP	Bone Gamma-Carboxyglutamate Protein (Osteocalcin)	ACACTCCTCGCCCTATTGGC	GCACCTTTGCTGGACTCTGC
COL1A1	Collagen, type 1, alpha 1	CTGCCTGGTGAGAGAGGTCG	GGACCTTCAGAGCCTCGGG
SPP1	Secreted phosphoprotein 1 (Osteopontin)	GGCCGAGGTGATAGTGTGGT	CCACGGCTGTCCCAATCAGA
RUNX2	Runt-related transcription factor 2	CAGCCACCGAGACCAACAGA	GCTGACATGGTGTCACTGTGC
SP7	Sp7 Transcription Factor (Osterix)	CCTGAGTGGAACAGGAGTGGA	AGGGAGGACGCCATCCTGAG
18S	18S rRNA	CTTAGAGGGACAAGTGGCG	ACGCTGAGCCAGTCAGTGTA

Primers were designed using the 'Pick primers' tool on the NCBI Nucleotide database and validated using in silico PCR on UCSC against the GRCh37/hg19 Human Feb. 2009 Assembly.

Supplementary Table 2. Candidates screened for effects on the hFOb cell line.

Candidate	Source	Dose
**LY294004**	Cell Signalling technology #9901	1.4 µM
**U0126**	Cell Signalling technology #9903	0.5 µM
**SP600125**	Sigma-Aldrich #S5567	40 nM
**Losartan potassium**	Sigma-Aldrich #SML3269	20 nM
**GF 109203 X**	Cell Signalling technology #37969	20 nM
**Rapamycin**	Cambridge bioscience #r0161	1 nM
**Calcitriol**	Sigma-Aldrich #D1530	500 nM

**Leukotriene D4**	Cambridge bioscience #CAY20310	1 pM
**17-β-estradiol**	Sigma-Aldrich #E8875	10 nM
**4-OHT**	Sigma-Aldrich #H6278	10 μM
**D-AP5**	Sigma-Aldrich #A8054	0.46 μM
**Kainic acid**	Tocris #7065	7.4 μM
